# Risk of visual axis opacification in infants with and without primary IOL implantation after congenital cataract surgery performed during the first 4 months of age

**DOI:** 10.1007/s00417-023-06143-9

**Published:** 2023-06-17

**Authors:** Martin Hložánek, Lenka Cilečková, Jorge L. Alió, Rudolf Autrata, Nina Zelenayová, Martin Komínek, Jiří Cendelín, Gabriela Mahelková

**Affiliations:** 1 Department of Ophthalmology for Children and Adults, Second Faculty of Medicine, Charles University and Motol University Hospital, V Úvalu 84, 15006 Prague 5, Czech Republic; 2 Department of Pathology and Surgery (Ophthalmology), Faculty of Medicine, University Miguel Hernandez, Avenida de la Universidad, s/n, 03202 Elche, Alicante, Spain; 3 Vissum Miranza Instituto Oftalmologico de Alicante, C/Cabañal, 1, 03016 Alicante, Spain; 4 Department of Paediatric Ophthalmology, Faculty of Medicine, Masaryk University and University Hospital Brno, Černopolní 9, 62500 Brno, Czech Republic

**Keywords:** Congenital cataract, Infants, Pupillary membrane, Posterior visual axis opacification, Intraocular lens, Aphakia

## Abstract

**Purpose:**

The study evaluates the rate of postoperative formation of a pupillary membrane (PM) and posterior visual axis opacification (PVAO) in infants with and without primary IOL implantation during the first 4 months of infancy.

**Methods:**

Medical records for 144 eyes (101 infants) operated between 2005 and 2014 were evaluated. A posterior capsulectomy and anterior vitrectomy were performed. Primary IOL implantation was performed in 68 eyes, while 76 eyes were left aphakic. There were 16 bilateral cases in the pseudophakic group and 27 in the aphakic group. The follow-up period was 54.3 ± 21.05 months and 49.1 ± 18.60 months, respectively. Fisher’s exact test was used for statistical analysis. The two-sample *t*-test with equal variance was used to compare surgery age, follow-up period and time intervals of complications.

**Results:**

The mean age of surgery was 2.1 ± 0.85 months in the pseudophakic and 2.2 ± 1.01 months in the aphakic group. PM was diagnosed in 40% pseudophakic and 7% aphakic eyes. A second surgery for PVAO was performed in 72% pseudophakic and 16% aphakic eyes. Both were significantly higher in the pseudophakic group. In the pseudophakic group, the number of PVAO was significantly higher in infants operated before 8 weeks of age compared to surgery age 9–16 weeks. The frequency of PM was not age-dependent.

**Conclusion:**

Although it remains feasible to implant an IOL during the primary surgery, even in very young infants, there should always be solid arguments for this decision since it puts the child at higher risk of repeated surgeries under general anaesthesia.
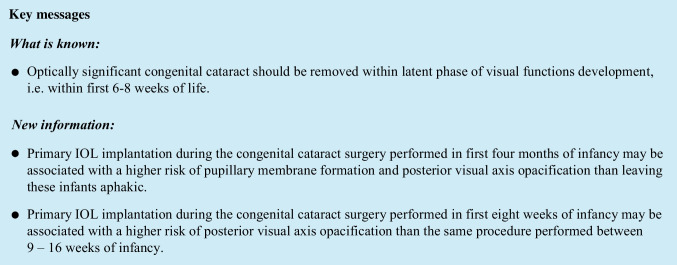

## Background

Congenital cataracts are a significant cause of preventable blindness and visual impairment in children, even in high-income countries [1, 2]. Early diagnosis and prompt surgical interventions are critical to prevent irreversible amblyopia [2]. Moreover, cataract extraction in an infant is not a static event. It is performed in the proinflammatory environment of the infant’s eye, during the period of its most fundamental growth and development [3]. It can have a significant impact on ocular structures and their subsequent growth [4]. Therefore, treatment of congenital cataracts in infants is a long-term process. It involves an early diagnosis, adequate timing of surgical treatment of visually significant cataracts, monitoring of visual functions and refractive errors with accurate optical correction and treatment of amblyopia and adequate treatment of any postoperative complications.

Primary implantation of artificial intraocular lens (IOL) in infants before 6 months of age is still controversial. It offers a great chance for in-the-bag implantation and permanent correction of the major part of aphakic refractive error at the time of surgery and, therefore, reduces or eliminates dependence on the use of contact lenses. On the other hand, there is a higher risk of intraoperative complications and poor predictions regarding the final refraction and the risk of inadequate myopic shift over time. Additionally, there is an increased risk of postoperative complications, including visual axis opacification.

The maintenance of a clear visual axis is critical for a good postoperative visual outcome after paediatric cataract extraction [2].

## Materials and methods

The aim of this retrospective chart review study was to evaluate the risk of two main postoperative complications that can lead to visual axis obscuration and additional surgery: pupillary membrane (PM) formation and posterior visual axis opacification (PVAO) in infants with and without a primary IOL implantation during the first 4 months of infancy. We retrospectively evaluated 144 eyes of 101 infants who have consecutively undergone surgery for a congenital cataract during the first 4 months of infancy (minimal age was 4 weeks and maximal age was 16 weeks) in the Department of Ophthalmology for Children and Adults of Motol University Hospital in Prague, between the years 2005 and 2014. Patients with the signs of congenital uveitis were excluded (corneal precipitates, posterior synechiae), as well as eyes with corneal diameter < 9 mm and eyes with secondary IOL implantation. The minimal follow-up period was 24 months. The study was approved by an institutional review board, performed following the tenets of the 1964 Helsinki Declaration and subsequent amendments, and the legal guardians of all patients signed written informed consent prior to surgery. Cataract removal was performed by 3 different surgeons, and the unified surgical protocol was followed during the whole period. Surgery was performed through a corneo-limbal incision, followed by a manual anterior continuous circular capsulectomy. Trypan blue was used for better visualization in cases of the poor red reflex. The lens material was aspirated using a coaxial or bimanual irrigation/aspiration system. A manual posterior circular capsulectomy (PCCC) with forceps and anterior vitrectomy (AV) using vitrector, through a corneo-limbal incision, were performed in all cases. The PCCC was at least 4 mm in diameter, which was 1 mm narrower in diameter than the anterior capsulectomy. Contact ultrasound biometry was performed right before the surgery, SRK/T formula was used for IOL calculation and the calculated power was reduced by 20%. The decision for IOL implantation was individual. Appropriate intraocular conditions for IOL placement and socioeconomic factors of patient’s parents (for example their visual acuity) played the main role in this decision, which was done during the surgery. All implanted IOLs were single-piece hydrophobic acrylic lenses (AcrySof SA60AT, Alcon, USA) and were implanted within the capsular bag; no capsular tension rings were used. Corneal incision and paracenteses were sutured using nylon 10/0, and stitches were removed 3 months after the surgery. No simultaneous bilateral surgeries were performed. All children were treated with the same perioperative and postoperative protocol. Subconjunctival dexamethasone 0.5 ml was administered at the end of surgery. Postoperatively, all patients were treated with dexamethasone 0.1% eye drops every hour for 6 days, followed by a slow taper over the next month. Fluorometholone was continued twice daily for another month. Homatropine 1% t.i.d. was prescribed for 2 weeks. All patients were routinely checked under the general anaesthesia in at least 3-month intervals, unless earlier examination was indicated. Outpatient visits were executed since adequate patient’s cooperation. Detailed microscopic examination of anterior and posterior segment was performed, as well as the measurements of the intraocular pressure (Tono-Pen® XL, Reichert, USA), objective refraction and the corneal diameter and curvature.

**Fig. 1:**
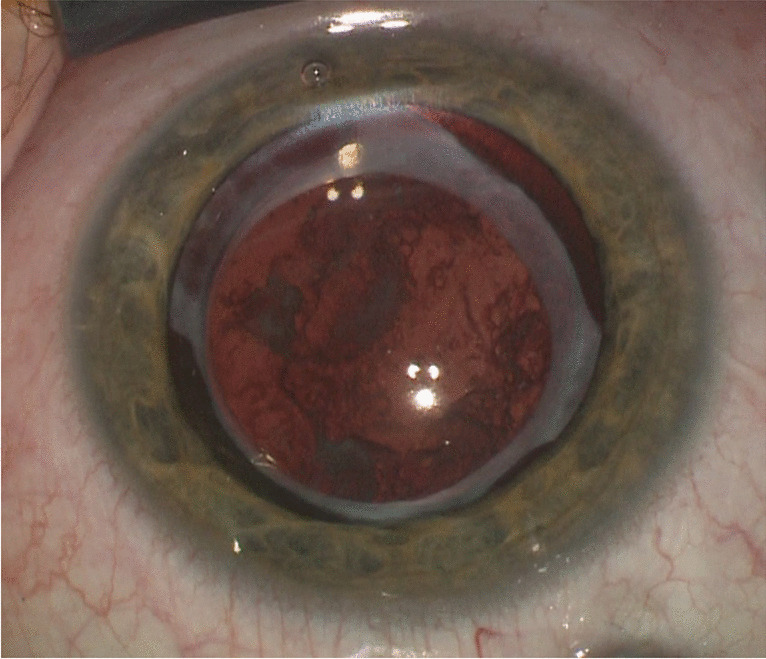
Lens cell proliferation extending into the pupillary space behind the implanted artificial intraocular lens

PM was defined as fibrous tissue located in front of the pupil, and PVAO was defined as lens cell proliferation extending into the pupillary space (Fig. 1). Both complications were considered relevant, either by obscuring the details of fundus examination or obscuring the retinoscopic reflex; both required surgery interventions. We evaluated the rate of PM and PVAO in two groups, i.e. with and without primary IOL implantation. Regarding the age of the primary cataract surgery, we divided both groups into two subgroups: surgery during weeks 0–8 and weeks 9–16. We compared the frequency of PM and PVAO between these subgroups within both the pseudophakic and aphakic groups. Since we compared nominal variables between groups of different populations, we used Fisher’s exact test for statistical analysis. The two-sample *t*-test with equal variance was used to compare surgery age, follow-up period and time intervals of complications diagnosed between groups. Results are expressed as mean ± SD; *p*-values less than 0.05 were considered statistically significant.

## Results

Primary IOL implantation was performed in 68 eyes of 52 children (22 boys, 30 girls), while 76 eyes of 49 children (26 boys, 23 girls) were left aphakic. There were 36 unilateral and 16 bilateral cases in the pseudophakic group, the age of surgery was 2.1 ± 0.85 months, and the follow-up period was 54.3 ± 21.05 months. In the aphakic group, 22 cases were unilateral, and 27 were bilateral, the age of surgery was 2.2 ± 1.01 months, and follow-up was 49.1 ± 18.60 months (Table 1). Differences between the two groups relative to age at the time of surgery and follow-up period were not statistically significant (*p* = 0.58, *p* = 0.12, respectively, 2 sample *t*-test). No significant difference in corneal diameter at the time of the surgery between the two groups was found as well (*p* = 0.14, 2 sample *t*-test).

**Table 1:** Characteristics of the cohort

	*N* (eyes)	Unilateral (patients)	Bilateral (patients)	Mean/median age of surgery (months)	Mean/median follow-up period (months)
Primary IOL implantation	68	36	16	2.1/2.0	54.3/53.5
Aphakia	76	22	27	2.2/2.0	49.1/45.0

PM was diagnosed in 27 eyes (39.7%) in the pseudophakic group. The secondary surgery was performed 4.78 ± 3.37 months after the primary surgery (range 2–20). In the aphakic group, a secondary surgery for PM was required in five eyes (6.6%). The interval was 4.15 ± 3.11 months after the primary surgery (range 1–9). Additional surgery for PVAO was performed in 49 pseudophakic eyes (72%); the interval between the primary and secondary surgery was 5.42 ± 3.69 months (range 2–19). In the aphakic group, surgery for PVAO was performed in 12 eyes (15.8%); the interval between the primary and secondary surgery was 5.42 ± 3.07 months (range 2–14). The number of PM and PVAO was statistically significantly higher in the pseudophakic than the aphakic group (*p* = 1.3exp-06, *p* = 4.0exp-12 respectively, Fisher’s exact test). The frequencies of PM and PVAO in the two subgroups (regarding surgery age) for both the pseudophakic and aphakic groups are shown in Tables 2 and 3.

**Table 2:** Occurrence of pupillary membrane (PM) formation and posterior visual axis opacification (PVAO) in the two pseudophakic subgroups

Pseudophakic group	Number of eyes	PM	PVAO
Whole group	68 (100%)	27 (39.7%)	49 (72%)
Surgery 0–8 weeks of age	44 (65%)	20 (45%)	37 (84%)
Surgery 9–16 weeks of age	24 (35%)	7 (29%)	12 (50%)
*p*		0.146	0.004

**Table 3:** Occurrence of pupillary membrane (PM) formation and posterior visual axis opacification (PVAO) in the two aphakic subgroups

Aphakic group	Number of eyes	PM	PVAO
Whole group	76 (100%)	5 (6.6%)	12 (15.8%)
Surgery 0–8 weeks of age	47 (62%)	3 (6%)	10 (21%)
Surgery 9–16 weeks of age	29 (38%)	2 (7%)	2 (7%)
*p*		0.719	0.086

In the pseudophakic group, the number of PVAO was significantly higher in the group of children who underwent surgery at an earlier age (*p* = 0.0037, Fisher’s exact test). In the aphakic group, the difference was not statistically significant. However, a similar trend could be observed (*p* = 0.0857 Fisher’s exact test). The difference in frequency of PM was not statistically significant within the groups (*p* = 0.146 in the pseudophakic group and *p* = 0.719 in the aphakic group, Fisher’s exact test).

## Discussion

In our study, we evaluated the risk of the postoperative visual axis obscuration, comparing two possible approaches of the primary aphakia correction. We concentrated on the specific group of patients in terms of the timing of primary surgical intervention in the age of 4–16 weeks.

The main aim of the congenital cataract surgery is to secure the clear visual axis. Furthermore, the proper timing of the surgery and the adequate aphakia correction are some of the crucial demands to provide the best possible conditions for the proper development of visual functions [2, 4–8]. Delayed surgery in case of the optically significant lens opacification can lead to the irreversible deprivation amblyopia and poor development of the central fixation with its consequences as nystagmus or strabismus. On the other hand, early cataract surgery can lead to a higher risk of postoperative complications [9–11].

Despite improvements in microsurgical techniques for cataract surgery, the development of secondary membranes and proliferation of lens material remains a major complication that often requires additional surgical procedures and hinders good visual outcomes in infants [2, 11, 12]. The risk of residual lens epithelial cell proliferation and PVAO development is reduced by performing a PCCC with an AV [9]; however, regrowth of the lens material is still possible. Development of PVAO after congenital cataract extraction with a PCCC (5 mm in diameter) and AV has been reported and showed that lens epithelial cells can proliferate even without the posterior lens capsule and anterior hyaloid support [13]. It was suggested that a larger posterior capsulectomy and a more extensive vitrectomy might prevent the formation of a secondary cataract [14]. Nonetheless, the PVAO is still the most common complication of paediatric cataract surgery and a reason for an additional surgery indication [12].

Many of the published results of the incidence PM and PVAO are consistent with our findings [8, 10, 15, 16].

Kuhli-Hattenbach et al. reported in 2008 the incidence of PVAO in aphakic eyes with primary surgery before 18 months of age was 9.2% [10]. Plager et al. also described very similar results. The primary surgery age in the latter study was comparable to our study groups and ranged from 3 weeks to 5 months (mean 2.5 months). The authors observed that 12.1% in the aphakic group required a second procedure for PVAO compared to 80.0% in the pseudophakic group [16].

On the other hand, Vasavada et al. reported PVAO in only four eyes (10.8%) in children with primary hydrophobic IOL implantation combined with PCCC and AV. Trivedi et al. reported visual axis opacification in infants with the same primary procedure in 23.6% of cases and Sukhija et al. in 13% of cases [17–19]. However, the mean age of the primary surgery in these reports was higher than in our study group (Vasavada 4.8 ± 2.4 months, Trivedi 6.0 ± 3.2 months and Sukhija 7.13 ± 2.32 months).

Lundvall and Zetterström observed ‘after-cataracts’ in 38.6% of infants without IOL implantation, with PCCC and AV; the mean age of surgery was 3.25 months [7]. The term ‘after-cataract’ was defined by the authors to include both PM and PVAO. The incidence of these complications was higher compared to our study. This could be partly explained by much lower doses of postoperative local steroids in the Swedish study (i.e. dexamethasone t.i.d. for the 1st week, b.i.d. for the 2nd and 3rd weeks and q.d. during the 4th week).

Solebo et al. published results of congenital cataract surgery in children during the first 2 years of life [3]. In groups with a primary IOL implantation, visual axis opacification (lens proliferation into the axis or inflammatory/pupillary membrane across the axis or capsular phimosis) was observed in 37% of bilateral cataracts and 42% of unilateral cataracts. In groups with primary aphakia, the same complication was reported in 13% of bilateral cataracts and 26% of unilateral cataracts. Data from that study were collected for 12 months postoperatively. This shorter follow-up period could have led to different complication frequencies compared to our pseudophakic group. However, the median interval between the primary and secondary surgery (3.9 months) was similar to our data but the 1-year follow-up period was much shorter than the follow-up in our study. Also, the mean age of primary surgery in groups with a primary IOL implantation was greater than in our study (13 weeks in bilateral and 35 weeks in unilateral cataracts). Interestingly, it was also much higher than the mean age of primary surgery in aphakic groups of the same study (7 weeks in both bilateral and unilateral cataracts). This fact could potentially lead to a reduction in complication rate in the pseudophakic group. The complication rate in our pseudophakic subgroup with surgeries performed at higher ages (i.e. 9–16 weeks) was similar.

Trivedi et al. described opacification of the visual axis requiring a secondary surgical procedure in 37.9% of eyes (11 of 29 pseudophakic eyes, mean age of primary surgery 4.8 ± 3.7 months). However, the rate was statistically significantly higher in infants when the primary surgery age was under 6 months of age (50% vs. 18.2% in infants undergoing surgery at ages greater than 6 months) [20]. Thus, the data may correspond with our observation of a higher incidence of PVAO in children undergoing primary IOL implantation at younger ages.

In the Infant Aphakia Treatment Study (IATS), more complications were observed in pseudophakic eyes compared to aphakic eyes during the first 5 years after surgery, and most complications occurred during the first year. PVAO (40%) and PM formation (28%) were the most common complications in the pseudophakic group, while the rates were low in the aphakic group (both 4%). We observed a higher incidence of PVAO in the pseudophakic and aphakic groups (72% and 16%, respectively) in our study. The incidence of PM was higher in the pseudophakic group (40%) in our study, while the incidence of PM in the aphakic group was similar (7%) [11].

In the Toddler Aphakia and Pseudophakia Study (TAPS), 178 eyes (96 children) were evaluated. TAPS is a registry of children treated by surgeons at the 10 centres who participated in the IATS. As in our study, the median surgery age was 2.5 months (range 1–7 months), and visual axis opacification including pupillary membranes was more common in pseudophakic (32%) than aphakic (8%) eyes, and adverse events, in general, were associated with younger age and smaller corneas. Visual outcomes were not affected by aphakia management [21].

At a time when the safety of cataract surgery with IOL implantation in adults was regarded as safe (15 years ago), one of several controversies was whether primary IOL implantation in infants with congenital cataracts was acceptable. Despite multiple problems with the fitting of aphakic contact lenses, many surgeons were enthusiastic about primary IOL implantation and believed that it could improve visual outcomes [8, 15]. It was generally accepted that congenital cataract surgery should be performed in the first 4–6 weeks of life in cases of unilateral cataracts, and during the first 6–8 weeks of life in cases of bilateral cataracts, to prevent the development of stimulus deprivation amblyopia, strabismus and nystagmus [2, 5, 6]. However, most publications report outcomes of cataract surgery at much higher ages, usually in children between 6 and 12 months. Our study concentrated on younger infants and found that even very small differences in the age of surgery (range 0–8 weeks vs. 9–16 weeks) increased the risks of complications in the pseudophakic group and increased the risks of PVAO in the aphakic group. This may also explain why we observed more complications in infants with IOL implantations compared to some other reports.

There was higher proportion of monocular cataracts in the group with primary IOL implantation in our cohort. This fact could theoretically be related to a higher number of eyes with persistent foetal vasculature (PFV). As this condition could predispose to PVAO, we subsequently inspected both groups for PFV. There were 9 eyes (13%) with PFV in pseudophakic group and 16 eyes (21%) with PFV in aphakic group. Therefore, higher number of PVAO in pseudophakic group in our cohort should not be related to the presence of PFV. We did not evaluate the visual outcomes in our study. However, according to published data, visual outcomes of infants with primary IOL implantation remain at least comparable with the aphakic groups in the majority of reports [8, 15, 22].

## Conclusions

In our study, we concentrated on infants operated during the age period recommended for intervention in case of significant congenital cataract. The rate of both PM and PVAO formation was higher in infants with primary IOL implantation (39.7% vs 6.6% and 72% vs 15.8%, respectively) in our study. Performing the surgery during the first 8 weeks of infancy was associated with a higher risk of PVAO in the pseudophakic group and similar trend was observed in the aphakic group, compared to infants operated between 9 and 16 weeks of life. These results suggest that although it remains feasible to implant an IOL during the primary surgery, even in very young infants, there should always be solid arguments for this decision since it puts the child at higher risk of repeated surgeries under general anaesthesia.

## Data Availability

The datasets used and analysed during the current study are available from the corresponding author on reasonable request.
